# Epidemiology of brain tumors in the United Arab Emirates: a National Registry Cross-sectional Study

**DOI:** 10.1186/s12883-020-01869-z

**Published:** 2020-08-14

**Authors:** Sarah Khan, Mona El Kouatly Kambris, Eman T AlShamsi

**Affiliations:** 1 College of Natural and Health Sciences, Zayed University, Dubai, United Arab Emirates; 2 Tawam Hospital, Abu Dhabi, United Arab Emirates

**Keywords:** Brain tumors, Epidemiology, Incidence rate, United Arab Emirates

## Abstract

**Background:**

Cancer is the third leading cause of death in the United Arab Emirates (UAE); brain cancer ranks 10th among the cancers, with 2.9% of the primary cancers originating from the nervous system. The epidemiology of brain cancers has not been explored. The unique population dynamics of UAE make it a fertile ground for analyzing the epidemiology of brain cancer. In this study, we aim to look at the frequency patterns and distribution of malignant primary brain tumors in the UAE.

**Methods:**

A cross sectional study was carried out using data obtained from the Tawam Hospital Cancer Registry for the years 1984–2017. The sample size included 756 diagnosed cases of malignant primary brain tumors in the UAE. Using SPSS and Excel software, frequencies, mean ages, histological type frequencies, average annual crude incidence rates and average annual age adjusted incidence rates were analyzed.

**Results:**

The expatriate population had higher percentage of brain tumors (72%) than the locals. The mean age at diagnosis was 33.48 years (± 21.14 years) with a male to female ratio of 1.69. Diffuse astrocytic and oligodendroglial tumors were the most commonly diagnosed tumors overall. Older adults had more cases of lymphoma while embryonal and ependymal tumors were most commonly seen in younger age groups. The overall average annual crude incidence rate for 2013–2016 for all primary brain tumors was 0.56 per 100,000 population.

**Conclusion:**

This is the first cancer registry study in the UAE that describes histological types of primary brain tumors based on the WHO 2016 classification of brain tumors and highlights their incidence rates. Through this study, several patterns of incidence trends for brain tumors in the UAE, according to histological types, sex and age groups have been recognized. Comparative studies would help identify the influence of potential changes in lifestyle, environmental or occupational risk factors on primary brain tumors.

## Background

Primary central nervous system (CNS) tumors include a complex, diverse group of benign and malignant tumors originating from the brain and its surrounding structures. More than 90% of the primary CNS tumors arise in the brain, the remainder are observed in the meninges, spinal cord and cranial nerves [1]. There are more than a hundred histologic subtypes of primary brain tumors [2]. Primary brain tumors are relatively rare, particularly in adults; however, they are the second highest cause of cancer mortality in people under 19 years of age [2]. Primary brain tumors may range from being benign to aggressive; nevertheless, even benign tumors are potentially lethal. Malignant brain tumors represent 1.4% of all cancers and 2.4% of all cancer mortality [3].

Brain tumors are somewhat uncommon considering one individual in 165 will be diagnosed with a brain tumor in their lifetime [4]. However, trends from 1970s onwards have shown an increased diagnosis of brain tumors in developed countries, with a world age standardized incidence rate that ranges from 4.3 to 18.6 per 100,000 per year [2, 5]. Gliomas are the most common histological type of primary CNS tumors [1]. Increasing incidence of brain tumors has been attributed to improved ability to diagnose brain tumors using computerized tomography scanning and magnetic resonance imaging [2].

Given the difference of incidence rates over geographical regions, race and sex, there is a need for greater understanding of the environmental etiology and risk factors of brain tumors [1], particularly those factors that are not accounted for by variations in reporting, diagnostic methods and access to health care [4]. In general, the incidence rate of malignant brain tumors is higher in men compared to women; however, women have a higher prevalence rate, indicating a possibly better survival rate among women [4–6]. Other than ionizing radiation, only a few risk factors have been consistently associated with brain tumors. Possible risk factors include smoking, alcohol consumption, mobile phone usage, industrial exposures, infectious agents and some rare genetic associations. Study of distribution over geographic regions may help in understanding the role of extrinsic factors in the distribution of brain tumors [1, 4].

Intracranial tumors can be classified in several ways. The most basic classification would depend on whether the tumors are primary or metastatic in origin. Primary intracranial tumors can further be described in accordance with the region they originate from; such as brain parenchyma, pituitary, pineal region, meninges, or the base of the skull. However, the World Health Organization (WHO) classification is considered the most apt for diagnostic purposes [7]. The WHO 2007 classification was based on histological features only, the more recent 2016 CNS tumor classification aims towards an integrated diagnosis that includes genotypic and phenotypic characteristics alongside microscopic findings [8, 9].

According to the World Bank statistics, the United Arab Emirates (UAE) had a population of 9.68 million in the year 2019, which makes up 0.13% of the world population. UAE comprises of seven emirates of which the most heavily populated emirate is Dubai with 35.7% of the population, followed by Abu Dhabi 34.7%, Sharjah 16.2%, Ajman 5.8%, Ras al Khaimah 4.1%, Fujairah 2.7% and Umm al Quwain 0.9% of the total population. Majority of the population falls into the age group of 25- 54 years, of whom a large part comprises of the expatriates. The median age of the population is 32.6 years with a male to female population ratio of 72 %: 28%. The greatest disparity in ratio of males to females is seen in the age group 25-54 years where there are 4.83 million males compared to 1.47 million females, this is mainly due to a larger number of expatriate male workers. The expatriate population comprises of 88.52% of the total UAE population. Indians comprise the majority of the expatriate population (27.49%), followed by Pakistanis (12.69%), while the remaining expatriate population comes primarily from Bangladesh, Philippines, Iran, Egypt, Nepal, Sri Lanka and China [10, 11].

Cancer is the third leading cause of death in the UAE following diseases of the circulatory system and injuries. The cancer incidence in 2015 was 54.5 per 100,000 population [12] with a mortality of about 500 deaths per year [13]. In the UAE, breast cancer topped the list of malignancies in 2016: 25.3% of cancers originated in the breast. Brain cancer ranked 10th with 2.9% of the primary cancers originating from the nervous system. In males, brain cancer ranked 9th with 3.9% of the malignancies in men originating from the brain whereas in women, brain cancer ranked 10th, comprising 2.5% of malignancies in women [12].

Tawam hospital, being the first cancer care facility in the UAE, became the first cancer referral hospital in the country in 1983. The first Tumor Registry in the UAE was compiled in 1983 as a hospital-based Tumor Registry at Tawam Hospital. Later, this registry was developed as the official UAE National Cancer Registry in 1998. The reporting of brain tumors is expected to be exhaustive since a Ministerial Decree made notification of cancer cases mandatory in UAE [13]. The data collected by the registry includes patient’s demographic details, tumor information, staging information, cancer treatment and follow up. A large majority of roughly 75-80 % of all cancer cases in the UAE are included in the Al Tawam cancer registry database as Tawam hospital, beside another hospital in Abu Dhabi, are the only two government facilities that offer all three forms of cancer treatment: surgery, radiation and chemotherapy [14–17].

Despite the plethora of data available from the cancer registry, very few studies explore the epidemiology of brain cancers in the UAE. There is significant geographical variation in the incidence of brain cancers around the world. This variation could be reflective of differences in diagnostic abilities and reporting practices; however, it is imperative that environmental and sociodemographic characteristics be explored to understand the patterns and risk factors associated with brain cancer. The UAE has unique population dynamics with almost 89% of the population comprising of expatriates which makes it a fertile ground for analyzing the epidemiology of brain cancer [11]. In this study we aim to look at the patterns of distribution and frequency of primary brain tumors in the UAE.

## Methods

### Study design and sample

A cross sectional study was carried out using secondary data obtained from the Tawam Hospital Cancer Registry. For this particular study, data on age, sex, residence, nationality, histological classification and year of diagnosis was obtained from the years 1984–2017. The analytic dataset included 756 diagnosed cases of malignant primary brain tumors in the UAE collected following establishment of the registry.

### Data analysis

Since data was obtained over a duration of 33 years, varying data collection protocols in a newly established registry translated into missing data for several data categories. Depending on the type of data missing, some analysis was done for the duration of 1984–2017 (756 cases), while some were only done from 2008 to 2017 (379) cases. All cases were coded following the International Classification of Diseases for Oncology, Third Edition (ICD-O-3). Following data cleaning, tumor histological codes were matched and grouped under the categorization highlighted in the WHO 2016 classification and then these groups were used to analyze the data. Any tumors with missing histologic confirmation or that were not classified under the ICD-O-3 site codes were removed from the data. All cases reported were from patients who had been seen in UAE hospitals and did not include autopsy diagnosed tumors. Due to the small number of cases, it was not possible to see trends while analyzing each histological type individually. Based on the diagnosed cases, the investigators identified primary brain tumors under 13 categories: 1. Diffuse astrocytic and oligodendroglial tumors (93,803, 93,823, 94,003, 94,013, 94,113, 94,403, 94,423, 94,503, 94,513, 94,603), 2. Other astrocytic tumors (94,213, 94,203), 3. Ependymal tumors (93,913, 93,923), 4. Choroid plexus tumors (93903), 5. Neuronal and mixed neuronal-glial tumors (95053), 6. Embryonal tumors (94,703,94,713, 94,733, 94,743, 94,903, 95,083), 7. Tumors of cranial and paraspinal nerves (95600), 8. Meningiomas (95,300, 95,303), 9. Mesenchymal, non-meningiothelial tumors, (91,503,92,203), 10. Melanocytic tumors (87203), 11. Lymphomas (95,913, 96,803, 96,903, 96,993, 97,143, 96,701, 96,753, 96,843, 96,873), 12. Germ cell tumors (90,643, 91,003),13. Other cancers (80,003, 92,503, 95,003, 97,313).

Using SPSS (version 26) and Excel software (version 16.37), counts, frequencies, mean ages, male to female sex ratios, proportions, rates, and other relevant statistics were calculated. Average annual crude incidence rates (AAIR) and 95% confidence intervals (95% CI) were estimated per 100,000 population, based on one-year age groupings. Population data for calculations was acquired from the World Bank data and Central Intelligence Fact Sheet [18, 19]. Several tables were inspired by representations in CBTRUS statistical report for primary brain and CNS tumors in the US for 2012-2016 [20]. To facilitate international comparison, the AAIR have been age-adjusted to the 2017 Gulf Cooperation Council (GCC) population (AIR^GCC^) and 2000 United States (US) standard population (AIR^US^). Direct method of standardization of AAIR was applied for years 2013-2017.

### Ethical approval and ethical considerations

Ethics approval for this study was obtained from the Tawam Human Research Ethics committee (T-HREC) in February 2018 (SA/AJ/559). Following ethical approval, data on primary brain tumors from the cancer registry was shared with the investigators. A second ethics approval was obtained from Zayed University Research Ethics Committee (ZU18_56_E). Ethics approval mandated that the ‘Good Clinical Practice’ and ‘Collaborative Institutional Training Initiative on Social and Behavioral Research’ was completed by the researchers.

The secondary data received from the cancer registry did not include the names of patients who had been diagnosed with brain cancers. However, to further ensure anonymity and respect privacy of the patients, the file number on record was also removed before data was analyzed. The researchers did not have access to any personal information that could be traced back to identify the patients.

## Results

Between 1984 and 2017, 756 cases of brain tumors were recorded. As the brain tumor cases were collected over a time period that exceeds three decades, it was expected to find missing values in some variables. Nationality and place of residence (i.e. Emirates) had some missing values. To overcome bias in data reporting, the frequency distribution for these two variables was limited to the time period of (2008-2017).

The expatriate population had higher percentage of brain tumors (72%) than the locals (Table 1). This distribution is consistent with the UAE demography, as local UAE nationals make up around 20% of the population according to the most recently available data [21]. However, the average annual incidence rate (per 100,000) of primary brain tumors was higher among UAE local population compared to expatriates (1.07: 0.35).

**Table 1:** Distribution of ten- year total, annual average total and average annual incidence rate (AAIR) of brain tumors by nationality, UAE, 2008–2017

Nationality	Ten-year total (%)	Annual average ^a^	AAIR per 100,000	95% CI
UAE	106 (28.0)	11	1.07	0.81 to 1.33
Expatriates	273 (72.0)	27	0.35	0.3 to 0.4
Total	379 (100.0).	38		

a. Annual average cases are calculated by dividing the ten-year total by 10

It was observed that majority of the patients diagnosed with malignant brain tumors were given first course of treatment in the UAE. Of those who were provided treatment within UAE, the majority were expatriate population (Tables 2 and 3) [10].

**Table 2:** Distribution of brain tumor cases by location of first course treatment (surgery, radiotherapy, and chemotherapy), UAE, 2008-2017

First Course Treatment Location			
	Surgery n (%)	Radiation n (%)	Chemotherapy n (%)
Inside UAE	58 (49.2%)	65 (55.1%)	55 (46.6%)
Outside UAE	40 (33.9%)	20 (16.9%)	13 (11%)
Combination	0 %	0 %	8 (6.8%)
No treatment	20 (16.9%)	33 (27.9%)	42 (35.6%)
Total	118 (100%)	118 (100%)	118 (100%)

**Table 3:** Distribution of treatment location (surgery, radiotherapy and chemotherapy) by nationality of brain tumor cases, UAE, 2008-2017

	UAE N (%)	Expat N (%)	*p*-value
Surgery within UAE	7 (12.1)	51 (87.9)	0.014
Radiotherapy within UAE	5 (7.7)	60 (92.3)	0.000
Chemotherapy within UAE	7 (7.3)	51 (92.7)	0.001

Regarding the distribution of brain tumors by Emirates, almost half of the brain tumor cases (46.4%) were living in Abu Dhabi, the capital of the UAE. In Dubai, the most populous city in the UAE, 18.4% of brain tumor cases resided. Moving further towards the North of the capital, one can notice a decreasing trend in the rate of occurrence of brain tumors (Table 4). This decreasing trend matches the UAE population density as around 70% of the population reside in Abu Dhabi and Dubai [10].

**Table 4:** Ten-year total, annual average total ^a^, and annual crude incidence rates (CIR)^b^ per 100,000 of brain tumors by Emirates, UAE

Period of inclusion: 2008-2017					
Emirate	Ten-year total	Annual average	% of all tumors	CIR 2005	CIR 2015
Abu Dhabi	134	13	46.4%	0.36	0.83
Dubai	55	5	18.4%	0.76	0.12
Sharjah	58	6	19.4%	0.63	0.64
Ras Al Khaimah	16	2	5.3%	1.99	0.68
Ajman	21	2	7.0%	0.48	0.00
Fujairah	9	1	3.0%	0.00	0.47
Umm Al Quwain	6	1	2.0%	0.00	0.00
Total^c^	299	30	100%	0.54	0.42

^a^: Annual average cases are calculated by dividing the total by ten.

^b^: CIR is calculated for years 2005 and 2015.

^c^: Excludes cases from outside UAE.

The crude incidence rate (CIR) in Abu Dhabi increased from 2005 to 2015, however decreased in Dubai and Ras Al Khaimah (Table 4).

The mean age at diagnosis was 33.48 years (± 21.14 years) and 201 patients (27.0%) were under 15 years of age when they were initially diagnosed. Most cases (46.7%) were in the age group of 25-54 years when diagnosed (Table 5).

**Table 5:** Distribution of Brain Tumors by Age at Diagnosis, UAE, 1984–2017

Age at Diagnosis	n (%)
0-14	201 (27.0)
15-24	60 (8.1)
25-54	348 (46.7)
55-64	80 (10.6)
65+	56 (7.5)
Total	745 (100.0)

Mean Age at Diagnosis: 33.48 years (± 21.14 years).

For the period (2008 – 2017), the highest annual crude incidence rate was reported for the year 2008. There is an overall decreasing trend in the annual CIR over the years (Table 6). The average annual CIR of brain tumors for the period 2008 – 2017 was 0.72 per 100,000 (95% CI: 0.60 to 0.86). A higher CIR in males as compared to females was seen only in 2010 and 2012.

**Table 6:** Crude Incidence Rate (CIR) of primary brain tumor cases by sex and year of diagnosis, UAE, 2008 – 2017

Year	Number of Male Cases	Number of Female Cases	Total Number of Cases	Male CIR per 100,000	Female CIR per 100,000	Male CIR /Female CIR	Total CIR per 100,000
2008	29	19	48	0.91	1.31	0.70	1.04
2009	28	16	44	0.85	1.07	0.80	0.92
2010	28	11	39	0.85	0.73	1.16	0.81
2011	25	20	45	0.71	1.24	0.57	0.87
2012	31	8	39	0.85	0.48	1.77	0.73
2013	22	13	35	0.59	0.76	0.77	0.64
2014	21	12	33	0.54	0.68	0.80	0.59
2015	22	17	39	0.56	0.94	0.59	0.67
2016	22	11	33	0.54	0.59	0.92	0.56
2017	13	11	24	0.31	0.57	0.54	0.40
**Total**	**241**	**138**	**379**	**6.71**	**8.37**	**0.80**	**7.23**
**Annual Average**^a^	**24**	**14**	**38**	**0.67**	**0.84**	**0.80**	**0.72**
**95% CI**				**[0.53, 0.81]**	**[0.63-1.04]**		**[0.60-0.86]**

^a^ Annual Average is calculated by dividing the total by ten.

Overall, high-grade tumors (WHO III/IV) constituted 48.1% (39.3 and 8.8%, respectively) of all cases while the rest were low grade tumors (WHO I/II) (4.1 and 11.8% respectively).

**Fig. 1:**
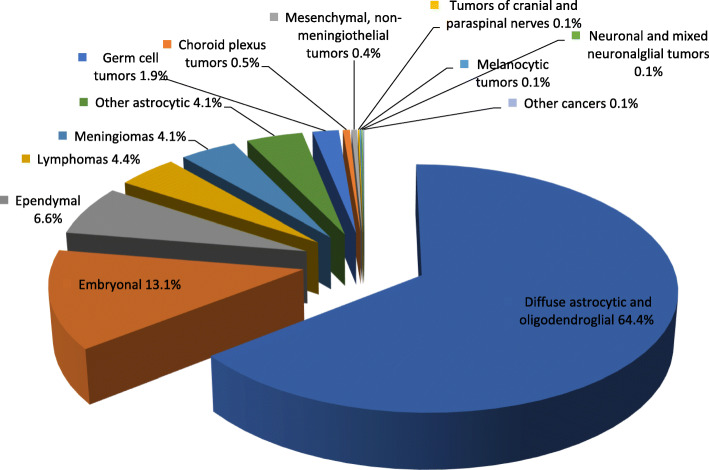
Distribution of brain tumors by histological type, UAE, 1984–2017

Figure 1 shows tumor distribution according to the histological classification group. Diffuse astrocytic and oligodendroglia accounted for 64.4% and embryonal tumors constituted 13.1% of the cases.

**Fig. 2:**
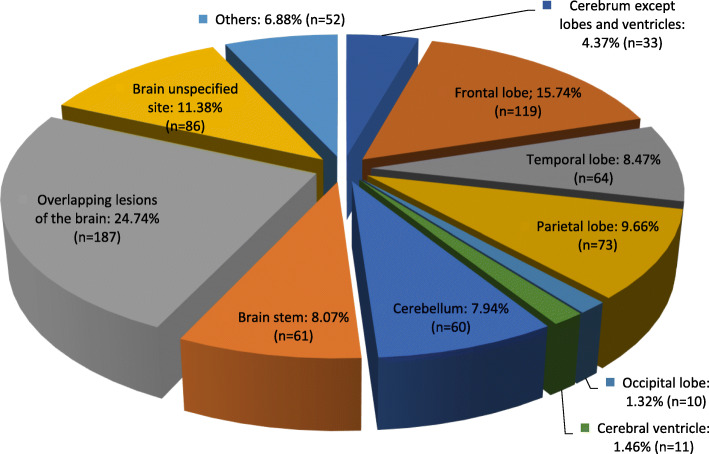
Distribution of brain tumors by tissue of origin, UAE, 1984–2017. (‘Others’ include CNS not otherwise specified, Tentorium not otherwise specified, Cerebral meninges, Dura not otherwise specified)

Figure 2 depicts the sites involved in the brain. More than 15 % of the tumors were located in the frontal lobe and another 10% were in the parietal lobe; around 25% (187 cases) involved overlapping lesions in the brain.

There were more male cases than females for most histological types, the highest being recorded for diffused astrocytic and oligodendroglial tumors (321 males vs 162 females). In addition, ependymal tumors occurred equally among both genders. Meningiomas and lymphomas were seen more commonly in older adults (mean age at diagnosis 48.0 and 49.5 years respectively) while the younger population saw more cases of embryonal and ependymal tumors (mean age at diagnosis 11.30 and 18.87 years respectively). The mean age distribution for males and females by histological type were almost similar for all types except for germ cell tumor and embryonal tumors. Females had germ cell tumors at an older age (mean age 33.50 years, ± 33.63 years) as compared to males (mean age at diagnosis 18.78 years, ± 24.74 years). A significant difference was found between the mean age distribution of males and females for embryonal tumors. Embryonal tumors occurred among males at an older age (mean age 13.2 years, ± 12.31 years) as compared to females (mean age at diagnosis 8.8 years, ± 7.83 years) (Table 7).

**Table 7:** Case distribution by histological type, with the number of cases, sex ratio, and mean ages at diagnosis by gender, UAE, 1984-2017^a^

Histology	n (%)	Male,n	Female, n	Sex Ratio	Mean ages	Mean age, (±SD) male	Mean age, (±SD) female
Diffuse astrocytic and oligodendroglial tumors	483(65%)	321	162	1.98	38.5	39.95 (±18.86)	37.60 (±19.75)
Other astrocytic	31(4%)	16	15	1.07	20.8	22.44 (±18.12)	19.13 (±15.87)
Ependymal	46(6%)	23	23	1.00	18.87	18.56 (±21.95)	19.17 (±18.64)
Embryonal*	97(13%)	55	42	1.31	11.30	13.20 (±12.31)	8.81 (±7.83)
Meningioma	31(4%)	17	14	1.2	48.03	50.06 (±12.60)	45.57 (±19.59)
Lymphoma	33(4%)	20	13	1.54	49.45	50.30 (±15.09)	48.15 (±12.08)
Germ cell tumors	13(2%)	9	4	2.25	23.31	18.78 (±24.74)	33.50 (±33.63)
Total^b^	744	467	277	1.69	33.48 (±21.14)	34.77 (±20.83)	31.31 (±21.52)

*: *p* < 0.05

^a^. Categories with fewer than 5 cases reported are not presented.

^b^. Refers to all brain tumors including types not presented in this table.

There were 161 incident primary brain tumor cases recorded in the registry during the period 2013-2017. The overall average annual crude incidence rate for 2013–2016 for all primary brain tumors was 0.56 per 100,000 population (Table 8). When standardized to the 2000 US standard population, the overall average annual incidence rate was 1.19 per 100,000 population. When standardized to the 2017 GCC population, the overall average annual incidence rate was 0.67 per 100,000 population. The average annual crude incidence rate was 0.69 per 100,000 for children aged 0-14 years, 0.33 per 100,000 population for those aged 15-24 years, 0.43 per 100,000 population for adults aged 25-54 years, 1.63 per 100,000 population for those aged 55-64 years, and 5.23 per 100,000 population for those over 65 years of age. Those aged 15-24 years had the lowest average annual age-adjusted incidence rates (both AIR^**GCC**^ and AIR^**US**^**)**. The highest average annual age-adjusted incidence rate was reported for those over 65 years of age. Age-adjusted incidence rates of embryonal and ependymal tumors were highest among children while age-adjusted incidence rates of diffuse astrocytic and oligodendroglial tumors were highest among those over 65 years of age (Table 8).

**Table 8:** Five-year total, average annual crude incidence rate with 95% confidence intervals by histological type, and average annual age-adjusted incidence rates^a^ with 95% confidence intervals, for all primary brain tumors, UAE, 2013-2017

	**Age at Diagnosis (0-14 Years)**						
**Histology**	**5-Year Total**	**IR**	**95% CI**	**AIR**^**GCC**^	**95% CI**	**AIR**^**US**^	**95% CI**
**Diffuse astrocytic & oligodendroglial**	14	0.24	[0.10, 0.38]	0.04	[0.018, 0.07]	0.05	[0.028, 0.08]
**Other astrocytic**	2	0.03	[-0.01, 0.07]	–	–	–	–
**Ependymal**	7	0.12	[0.05, 0.19]	0.02	[0.01, 0.03]	0.02	[0.002, 0.04]
**Embryonal**	17	0.29	[0.13, 0.44]	0.05	[0.024, 0.08]	0.06	[0.03, 0.09]
**Germ cell tumors**	1	0.02	[-0.01, 0.05]	–	–	–	–
**Subtotal**	**41**	**0.69**	**[0.34, 1.04]**	**0.13**	**[0.06, 0.19]**	**0.15**	**[0.07, 0.22]**
	**Age at Diagnosis (15-24)**						
**Histology**	**5-Year Total**	**IR**	**95% CI**	**AIR**^**GCC**^	**95% CI**	**AIR**^**US**^	**95% CI**
**Diffuse astrocytic & oligodendroglial**	8	0.2	[0.08, 0.30]	0.04	[0.01, 0.07]	0.03	[0.01, 0.05]
**Other astrocytic**	2	0.05	[-0.001, 0.11]	–	–	–	–
**Choroid plexus tumors**	1	0.03	[-0.02, 0.08]	–	–	–	–
**Embryonal**	1	0.03	[-0.02, 0.08]	–	–	–	–
**Meningiomas**	1	0.02	[-0.03, 0.07]	–	–	–	–
**Subtotal**	**13**	**0.33**	**[0.09, 0.57]**	**0.06**	**[0.02, 0.11]**	**0.05**	**[0.01, 0.08]**
	**Age at Diagnosis (25-54)**						
**Histology**	**5-Year Total**	**IR**	**95% CI**	**AIR**^**GCC**^	**95% CI**	**AIR**^**US**^	**95% CI**
**Diffuse astrocytic & oligodendroglial**	64	0.36	[0.29, 0.43]	0.2	[0.17, 0.23]	0.16	[0.13, 0.19]
**Other astrocytic**	1	0.006	[-0.005, 0.17]	–	–	–	–
**Ependymal**	3	0.02	[-0.001, 0.041]	–	–	–	–
**Embryonal**	4	0.02	[-0.001, 0.041]	–	–	–	–
**Lymphomas**	4	0.02	[-0.001, 0.041]	–	–	–	–
**Germ cell tumors**	1	0.006	[-0.005, 0.017]	–	–	–	–
**Subtotal**	**77**	**0.43**	**[0.3, 0,57]**	**0.24**	**[0.16, 0.31]**	**0.19**	**[0.13, 0.25]**
	**Age at Diagnosis (55-64)**						
**Histology**	**5-Year Total**	**IR**	**95% CI**	**AIR**^**GCC**^	**95% CI**	**AIR**^**US**^	**95% CI**
**Diffuse astrocytic & oligodendroglial**	14	1.5	[0.45,2.55]	0.08	[0.03, 0.13]	0.13	[0.04, 0.22]
**Lymphomas**	1	0.1	[-0.10, 0.30]	–	–	–	–
**Subtotal**	**15**	**1.63**	**[0.26, 3.00]**	**0.08**	**[0.01, 0.15]**	**0.14**	**[0.02, 0.26]**
	**Age at Diagnosis (65+ Years)**						
**Histology**	**5-Year Total**	**IR**	**95% CI**	**AIR**^**GCC**^	**95% CI**	**AIR**^**US**^	**95% CI**
**Diffuse astrocytic & oligodendroglial**	14	4.8	[3.26, 6.34]	0.15	[0.1, 0.2]	0.61	[0.41, 0.8]
**Lymphomas**	1	0.38	[-0.4, 1.12]	–	–	–	–
**Subtotal**	**15**	**5.23**	**[2.08, 8.34]**	**0.16**	**[0.07, 0.26]**	**0.66**	**[0.26, 1.06]**
**TOTAL**	**161**	**0.56**	**[0.46, 0.66]**	**0.67**	**[0.54, 0.81]**	**1.19**	**[0.82, 1.56]**

^a^. Rates are per 100,000 and are age-adjusted to the 2017 GCC population (AIR^GCC^) and 2000 US standard population (AIR^US^)

-- Average annual age-adjusted incidence rates with 95% confidence intervals are not presented when fewer than 5 cases were reported for the specific category

Tumor categories with zero cases per age group were excluded from the above table

## Discussion

The overall incidence of brain tumors worldwide has been reported to be increasing in the last few decades. This increase has been attributed mainly to better diagnostic and imaging techniques [22, 23]. While generally, there is an increase in diagnosis of malignant brain tumors in UAE, the increase in cases recorded does not show a consistently upward trend as indicated in other studies [22–24]. In this study the number of cases diagnosed, and the crude incidence rate peaked in 2008 (48 cases, 1.04 per 100,000) after which the cases have declined comparatively. This increase in 2008 could possibly be due to the rapid influx of expatriates resulting in a higher percentage of population growth in the UAE during this time period [11]. It is noticeable that the incidence of primary brain tumors in Abu Dhabi increased from 2005 to 2015 whereas it decreased in Dubai and Ras Al Khaimah. This could be explained by the establishment of the Dubai Hospital Tumor Registry in 2014, which may have diverted or delayed reporting of some cases at the Tawam Hospital Cancer Registry in Abu Dhabi [17]. The highest percentage of brain tumors were diagnosed at grade IV differentiation, which is reinforced further by the finding of a high number of gliomas recorded in the registry. Health insurance became mandatory for all residents of UAE thus healthcare services became affordable to all, which could also explain the higher number of confirmed cases diagnosed over time. With healthcare insurance for all, the likelihood of cases going undiagnosed may have decreased. The availability of good quality healthcare to all residents also explains why a large number of expatriates have sought first course of treatment within the UAE rather than other countries [25].

The mean age at diagnosis of malignant brain tumors in this study was 33.48 years. Weeks (2016) reported the median age of diagnosis of all brain tumors to be at 59 years in the USA [24] while Ostrom et al. (2019) reported the median age of diagnosis of all primary brain tumors and all other CNS tumors to be at 60 years [20]. Darlix et al. (2016) reported a mean age at diagnosis of 52.6 years, 51.9 for males and 53.3 years for females in France [26], while Tamimi et al. (2016) in a study carried out in Jordan reported a mean age of diagnosis of 39.2 years. The mean age of diagnosis in Jordan is comparable to the findings in the UAE, indicating similarities which could be associated with socio-geographical similarities [23]. The mean age of diagnosis needs to take in to account the consideration that this study included only malignant primary brain tumors and not all primary brain tumors. Looking at population pyramid structures of Jordan, UAE and France we could possibly attribute the lower median age of diagnosis of malignant brain tumors to the comparatively greater percentage of population in the 24-54-year age group in UAE. This inflated population group is mainly due to the presence of expatriate population, predominantly male expatriates, who have also been recorded as having a seemingly higher number of diagnosed malignant brain tumors compared to the local UAE population (Table 1). Jordan does not show a similar increase in population in the middle age year groups but shows a heavier dominance of younger population, which could be an explanation for more tumors being diagnosed at a younger age. On the contrary, France shows a stable to aging population with the greatest dominance of people in the 45-79-year age group, possibly explaining why median age of diagnosis is higher. These features of the population distributions in various countries may offer some hints into explaining the varying mean age of diagnosis across different countries [11, 27, 28].

In the UAE, more males were diagnosed with malignant brain tumors with a male to female ratio of 1.69, a total of 467 (62.76%) males and 277 (37.24%) females. Darlix et al. (2016) reported a male to female ratio of 0.86 in France [26] and Tamimi et al. (2016) also reported a higher male to female ratio of malignant tumors in Jordan. Generally, a higher male to female incidence rate ratio for malignant brain tumors is observed in several studies showing international comparisons [4, 6]. However, in this study despite seeing higher number of males being diagnosed with malignant brain tumors, on comparing the CIR of men and women it was seen that women have a higher annual average CIR (0.84 per 100,000) compared to men (0.67 per 100,000). The UAE has a much higher male population (69.12%) compared to women (30.88%) explaining the overall higher number of malignant brain tumors in males. However, Jordan and France both have almost equal gender distributions and the dominance of male to female ratio of diagnosis cannot be rationalized on the basis of population distributions [11, 27, 28].

Bondy et al. (2008) highlighted medulloblastoma (embryonal category) and gliomas (diffuse astrocytic and oligodendroglial category) as the most common types of pediatric tumors (less than 15 years) in Sweden. Ostrom et al. (2019) reported that the incidence rates of astrocytoma, germ cell tumors and embryonal tumors were higher in younger age group and decreased with advancing age. This study in the UAE also showed a predominance of embryonal and diffuse astrocytoma and oligodendroglial tumors in the pediatric age group. Glioma and meningioma were the most common tumors seen in adults in Sweden [6]. Jazayeri et al. (2013) highlighted astrocytomas and glioblastomas as being the most frequent malignant brain tumor in adults in Iran. It was also noticed that even though males had more malignant brain tumors, meningiomas were more common in females [29]. Interestingly, Jordan showed meningioma as the most frequently found tumor followed by glioblastoma and astrocytoma. Meningiomas were seen to be the most frequent malignant tumors in Jordan, United States, France and Korea. Once again meningiomas were most frequent in females while astrocytomas were most common in males [23, 30]. This female predominance of meningiomas has been linked to hormonal influence in women. Greater expression of progesterone receptors in meningiomas may explain increased frequency in females as compared to males. Similarly, women seem to have less gliomas during menarche and menopause as noted by the New York State Cancer Registry [22] .

In this study, meningiomas were not as commonly seen as in other studies, however this may be due to the inclusion of only malignant brain tumors in this study. Tumors under the classification of astrocytic and oligodendroglial tumors were the most frequently found tumors in both males and females. Since gliomas and astrocytomas were all grouped under this category together the numbers may seem exaggerated compared to other studies. Nevertheless, the trend of gliomas and astrocytomas being the most commonly found adult tumor was seen in UAE as in other studies [7]. Malignant meningiomas were much less frequent and followed after embryonal, ependymal tumors and lymphomas. The total number of meningiomas seen in UAE was more in males, however this could be explained by the heavy predominance of the male population in UAE that could be masking the true comparison. Among women, the most commonly found tumor was under the category of diffuse astrocytic and oligodendroglial tumors followed by embryonal tumors, ependymal, meningiomas and lymphomas. In men, a similar trend was seen; however, lymphomas were more frequent than meningiomas. Embryonal tumors were found at a significantly older age in males compared to females.

The average annual crude incidence rate in this study was calculated to be 0.56/100000 and average age adjusted incidence rate calculated in this study was 1.19/100,000 which is much lower in comparison to the overall incidence of 7.08/100000 reported by CBTRUS and the range of 2.1-5.8 /100,000 incidence rate of primary brain tumors observed in various studies [6, 20] . The calculated incidence rate also appears to be lower than the age adjusted incidence rate of the New Zealand Cancer Registry and Gironde Cancer Registry (6.7 and 7.4 per 100,000 respectively) [2]. The incidence rate of primary malignant brain tumors in Iran was recorded as 2.74/100000 which is lower than seen in other developed countries [29]. Age adjusted incidence rates were calculated to make the data comparable to other international studies exploring epidemiology of malignant brain tumors that typically standardize to the 2000 US standard population. This study calculated annual average age adjusted incidence rate incidence rate to be 0.67/100000 when adjusted to the GCC standard population. The GCC population was used to provide comparison with the local context.

The findings in this study need to be considered in the light of some strengths and limitations Some weaknesses and limitations encountered were that the number of tumors diagnosed was low making it necessary to group the tumors into the WHO main histological categories to be able to analyze them, rather than performing analysis on each histological subtype. Since the secondary data was collected over three decades, there is the likelihood that diagnostic procedures and classification criteria may have changed over time, however most analysis in this study have been conducted from 2008 onwards. Registry studies are considered generalizable with strong external validity since they include typical patients representing a heterogeneous population [31]. However, the population in the UAE is so dynamic that generalizability should be considered with caution. The study includes only histologically confirmed cases of malignant brain tumors as listed in the data registry and may have missed cases that were not diagnosed in the UAE or not diagnosed at all. There is also the possibility that some tumors may have been metastatic but reported as primary malignant tumors, as diagnostic and reporting techniques evolved [32]. However, since only tumors classified under ICD-O-3 codes were included in the analysis such erroneous inclusions were most likely avoided. Due to the dearth of epidemiological studies on brain tumors in the region, we were unable to compare the findings of this study to others conducted in similar sociodemographic settings.

To the best of our knowledge, this is the first-time data on brain tumors has been reported and analyzed in the UAE and in the GCC. This study adopts the latest WHO classification of tumors of the central nervous system and has encompassed data on malignant brain tumors collected over three decades. The data was provided by the reputed Tawam Cancer Registry and thus held in high regard in terms of accuracy and rigor of collection [32]. This study brings together essential data on descriptive epidemiology of primary malignant brain tumors which could form the basis for clinical population-based studies. Also, this study lays the foundation for comparison and benchmarking for future studies carried out on brain tumors in the region.

## Conclusion

This is the first national registry study in the UAE that describes histological types of primary brain tumors based on the WHO 2016 classification of brain tumors and highlights their incidence rates. This study has analyzed more than three decades of data obtained from the Cancer Registry at Tawam Hospital. Through this study several patterns of incidence trends for brain tumors in UAE, according to histological types, sex and age groups have been recognized. The incidence rates were standardized to allow comparability with the US and GCC populations. These trends suggest a socio-geographical influence on the incidence of brain tumors. There is a need for further epidemiological studies of brain tumors in the region, such comparative studies would help identify the influence of potential changes in lifestyle, environmental or occupational risk factors on primary brain tumors.

## Data Availability

The data that support the findings of this study are available from Tawam Hospital Cancer Registry, but restrictions apply to the availability of these data, which were used under approval for the current study, and so are not publicly available. Data are however available from the authors upon reasonable request and with permission of Tawam Hospital Cancer Registry.

## Abbreviations

AAIR: Average Annual Crude Incidence Rate
CBTRUS: Central Brain Tumor Registry of the United States
CIR: Crude Incidence rate
CNS: Central Nervous System
ICD-O-3: International Classification of Diseases for Oncology, Third Edition
IR: Incidence Rate
GCC: Gulf Cooperation Council
UAE: United Arab Emirates
US: United States
WHO: World Health Organization
